# Degree of Contamination of Gutta-Percha Points by *Staphylococcus aureus* (MRSA/MSSA) Strains

**DOI:** 10.3390/ijms25168566

**Published:** 2024-08-06

**Authors:** Ana Moura Teles, Cristina Pina, Inês Lopes Cardoso, Antea Tramontana, Miguel Cardoso, Ana Sofia Duarte, Maria Bartolomeu, Rita Noites

**Affiliations:** 1 Faculty of Dental Medicine, Universidade Católica Portuguesa, 3504-505 Viseu, Portugal; amteles@ucp.pt (A.M.T.); mabcardoso@ucp.pt (M.C.); asduarte@ucp.pt (A.S.D.); mbartolomeu@ucp.pt (M.B.); 2 Centre for Interdisciplinary Research in Health (CIIS), Universidade Católica Portuguesa, 3504-505 Viseu, Portugal; 3 FCS-UFP, Universidade Fernando Pessoa, Faculdade de Ciências da Saúde, 4200-150 Porto, Portugal; cpina@ufp.edu.pt (C.P.); mic@ufp.edu.pt (I.L.C.); antea.nog98@gmail.com (A.T.); 4 FP-I3ID, Instituto de Investigação, Inovação e Desenvolvimento, FP-BHS, Universidade Fernando Pessoa, Biomedical and Health Sciences, 4249-004 Porto, Portugal; 5 CINTESIS.UFP@RISE, Centro de Investigação em Tecnologias e Serviços de Saúde, Rede de Investigação em Saúde, Universidade Fernando Pessoa, 4200-450 Porto, Portugal

**Keywords:** *Staphylococcus aureus*, MRSA/MSSA, Gutta-Percha, contamination, endodontics

## Abstract

Methicillin-resistant *Staphylococcus aureus* (MRSA) is considered one of the most harmful bacteria to human health. Dentistry, like all healthcare disciplines, places great emphasis on preventing scenarios that may result in cross-infection. Although various tested and already used materials are suitable for filling the root canal system, Gutta-Percha (GP) remains the preferred and widely accepted gold standard. Objective: We performed an in vitro analysis of the contamination of GP points, regarding the strains of Methicillin-resistant (MRSA) and Methicillin-sensitive (MSSA) *Staphylococcus aureus*, using classical microbiology methods and molecular biology techniques. Methods: Gutta-Percha points of two different brands from opened packages (already in use for 1 month) were collected for analysis. The assessment involved incubating the GP points in Brain Heart Infusion (BHI) medium to detect microbial growth. Growing microorganisms were plated on a selective and differential chromogenic medium for MRSA/MSSA strains, and the identification of isolates was confirmed by Polymerase Chain Reaction (PCR). In the case of microbial growth, the GP point was submitted to a disinfection protocol. Results: From the 315 collected GP points, only 6 (1.9%) resulted in being positive for microbial growth. After confirmation by PCR, only one sample of the six GP points was contaminated by MRSA, and the remaining five were MSSA-contaminated. The disinfection protocol was effective in all contaminated GP points. Conclusions: The Gutta-Percha points from opened pre-sterilized packages showed a very low degree of contamination by MRSA/MSSA. However, the detection of MSSA and MRSA strains raises concerns about potential contamination in dental clinic environments, and this risk cannot be considered negligible.

## 1. Introduction

*Staphylococcus aureus* is a Gram-positive bacterium, commonly detected in the human skin and nasal regions [1,2,3]. Its role in the pathology of the oral cavity is not yet fully understood [4]. Over the years, *S. aureus* has been correlated with several non-physiological oral conditions, such as acute suppurative parotitis [5,6], angular cheilitis [7], and staphylococcal mucositis [8,9]. This bacterium has also been detected in oral infections such as jaw cysts [10] and orofacial abscesses [11,12,13].

Despite its presence in the oral cavity and its highly pathogenic potential [14,15,16], *S. aureus* manages to coexist harmlessly in the human environment thanks to the mechanism known as homeostasis, maintaining an innocuous equilibrium [17,18,19,20]. However, when this balance is disrupted, the situation known as dysbiosis is created. This is a sharp shift that can be directly related to systemic and metabolic diseases such as diabetes, cardiovascular disease, periodontitis, and obesity, among many others [21,22]. Oral dysbiosis is a condition influenced by various factors, reflecting its multifactorial nature. A commonly recognized cause is the use of broad-spectrum antibiotics that exhibit systemic action [23,24]. As an example, amoxicillin, a β-lactam antibiotic of the penicillin class, is one of the most widely used antibiotics in dentistry and, within its applications approved by the Food and Drug Administration (FDA), is precisely used against *Staphylococcus* spp. [25].

However, Methicillin-resistant *Staphylococcus aureus* (MRSA) strains exhibit multidrug resistance, not only in β-lactams (penicillin, methicillin, carbapenems, and cephalosporins) but also to tetracyclines and macrolides [26]. On the other hand, Methicillin-sensitive *Staphylococcus aureus* (MSSA) strains are sensitive to these antibiotics.

MRSA strains are one of the major causes of nosocomial infections, leading to high mortality [27,28,29]. According to the European Centre for Disease Prevention and Control (ECDC)’s annual report of the Priority Program of Infections and Resistances to Antimicrobials, Portugal is considered one of the countries with the highest MRSA percentage in Europe [30]. Although the risk of infection in dental clinics is lower than in hospitals, *S. aureus* strains are still responsible for a large number of pathologies at the oral and perioral level, making this bacterial strain a public health problem [26]. Antibiotic prescription in dentistry, whether for prophylactic or therapeutic purposes, accounts for approximately 10% of antibiotic prescriptions worldwide and is not always considered appropriate, leading to excessive or incorrect antibiotic use in dental practice [31].

Dentistry is extremely concerned and focused on avoiding situations that can lead to cross-infection. The aim of Non-Surgical Root Canal Treatment (NSRCT) is the prevention or handling of apical periodontitis through debridement and cleaning of the root canal system (RCS). In this way, the RCS must be cleaned, shaped, and disinfected at the highest possible level [32], before three-dimensional filling, to prevent or minimize any chances of infection or reinfection. The procedures are performed under aseptic conditions in order to avoid the re-penetration of microorganisms or metabolites they produce [32,33,34,35]. In fact, despite the absence of pulpal tissue, the RCS continues to communicate with the external environment by apical foramen, accessory root canals, and dentinal tubules. This surely implies that sealing must be assured tri-dimensionally [33].

Several tested materials suitable for filling the RCS are available, with Gutta-Percha (GP) being the gold standard. Despite the production of GP points in aseptic conditions and their potential antimicrobial properties, particularly due to the zinc oxide component [36], the risk of contamination remains present. Factors such as handling, aerosols, and physical sources during storage may contribute to contamination, and sterility cannot be guaranteed [37]. In fact, several studies have confirmed the presence of bacteria even in freshly opened packages [38,39,40].

The aim of this in vitro study was to investigate the potential MRSA and MSSA contamination of GP points sourced from commercially available packages that had been in use for one month in a clinical setting, by classical microbiology methods and molecular biology techniques.

## 2. Results

### 2.1. Evaluation of GP Points Contamination

From the 315 collected samples, the percentage of contaminated GP points (1.9%) was minimal compared to the uncontaminated ones (98.1%; Table 1).

### 2.2. Phenotypical Identification of S. aureus (MRSA/MSSA)

**Figure 1:**
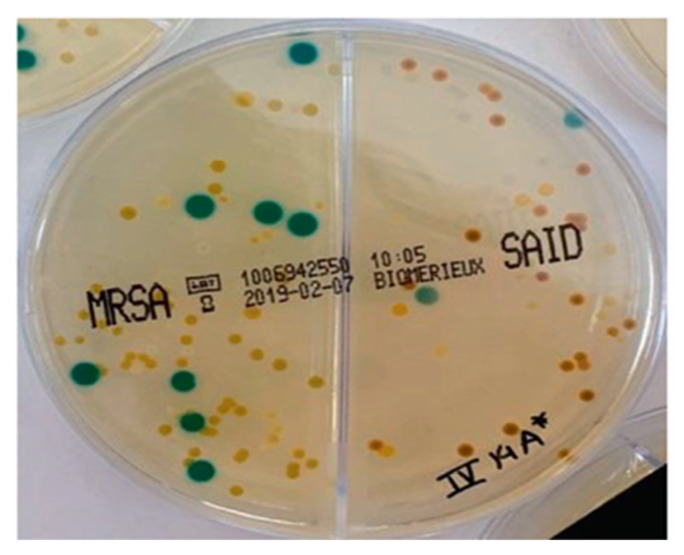
Typical colonies of MRSA and MSSA in chromID^®^ MRSA/chromID^®^ *S.aureus* (Biomérieux) —green is equivalent to MRSA, while beige corresponds to MSSA.

Following culture on the MSSA/MRSA selective medium, only one of the six positive samples (16.6%) exhibited a green phenotype, indicating the potential presence of MRSA. The remaining five samples (83.3%) displayed beige colonies, characteristic of MSSA (Figure 1).

All suspicious colonies exhibited a Gram-positive cocci morphology and tested positive for both catalase and coagulase, which are typical characteristics of *S. aureus* strains.

### 2.3. Molecular Identification of S. aureus (MRSA/MSSA)

**Figure 2:**
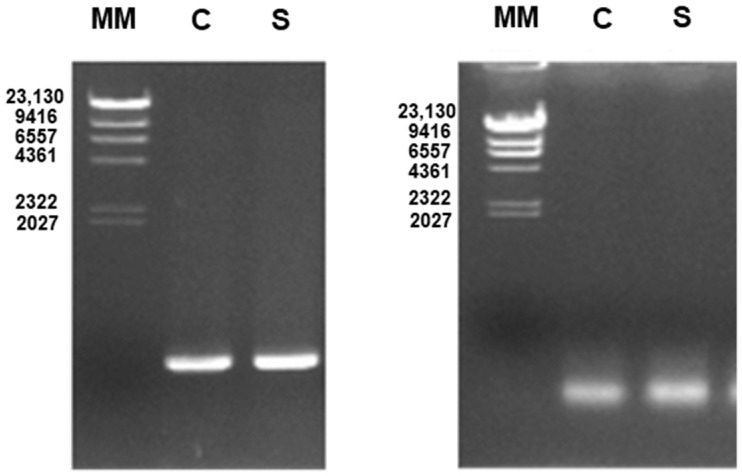
PCR products using primers for the *mecA* gene (527 bp; left image) and for the *nuc* gene (255 bp; right image). MM: molecular weight marker (lambda phage DNA cut with Hind III), with the length of the respective fragments (in base pairs): 23,130, 9416, 6557, 4361, 2322, and 2027. C: control MRSA (left image) or control MSSA (right image). S: positive sample for MRSA (left image) or MSSA (right image).

The identification of potential MRSA and MSSA strains was confirmed by PCR, where the amplification of the *mecA* gene confirmed MRSA and the detection of the *nuc* gene confirmed MSSA (Figure 2).

### 2.4. Chairside Disinfection Protocol (CPD)

As stated, microbial growth was detected in six tubes, each containing a single GP point. For disinfection purposes, the GP points were immersed in 3% NaOCl for 1 min, and disinfection was assessed after GP points were incubated in Brain Heart Infusion (BHI) medium at 37 °C for 72 h. No turbidity was observed, indicating that the chairside disinfection protocol effectively eliminated contamination in all six GP points (100%).

## 3. Discussion

Within the RCS, when NSRCT is performed, asepsis is impossible to be achieved due to anatomical irregularities. Smear-layer formation during the shaping phase of this treatment, which contains microorganisms and metabolites they produce, acts as a physical barrier, preventing the entrance of the irrigant solutions into dentinal tubules. To overcome this, some guidelines recommend that the correct cone selection should be confirmed by tug-back and periapical radiography, as well as the execution/application of a final irrigation protocol, to ensure a significant decrease of the microbial load inside the RCS, followed by three-dimensional filling of RCS, intending to create conditions compatible with the handling of periapical pathology of endodontic origin [33]. Nevertheless, according to Siqueira (2001), it is impossible to guarantee the total absence of microorganisms inside the RCS after an NSRCT.

NSRCT’s primary target is to create conditions aiming to prevent tooth loss; preserve, whenever possible, the restoration of the tooth; and provide adequate periodontal support. Otherwise, it becomes an absolute contraindication. An endodontic treated tooth must be functional, and NSRCT must prevent secondary endodontic infections.

The execution of NSRCT involves handling instruments and materials, as well as aerosol formation, which can pose significant risks of cross-contamination [41]. This risk can be challenging to manage if the dentist does not adhere to strict clinical protocols to prevent such contamination. In this way, there is a high risk of GP point contamination, which supported our investigation.

In fact, the prevention of cross-infection during an endodontic session relies on rubber dam use, disinfection of instruments, and attention to minimizing the exposure of GP packages [42]. A simple and accurate method for handling GP points is an assistant to hold the commercial package without touching the GP points. This allows the operator to use sterile tweezers (opened only by the operator at that moment) to carefully remove the desired point(s), ensuring that no materials, devices, or surfaces in the operative field are touched. This approach helps to minimize the risk of potential cross-infection.

Although GP points are sold as sterile and ready to use [43], careful handling is essential. When a GP point is removed from the commercial package, the remaining points are exposed, increasing the risk of cross-contamination during the filling of the pulpal space.

Nowadays, MRSA strains are endemic in several health units all over the world and, consequently, have become an important focus of global efforts on infection control. Due to the limited treatment options, these strains have become the highest cause of nosocomial infections worldwide, leading to high morbidity and mortality rates [27,28].

Taking into account the MRSA/MSSA potential contamination, in a dental office, the contact time between patients and staff is relatively short, as the transmission of MRSA is expected to be less problematic than in hospitals. Even so, cross-transmission of microorganisms in dental appointments—via direct contact (including blood–blood contact) or via the inhalation/ingestion of microorganisms present in bioaerosols from dental unit water [44]—remains an almost-unavoidable problem. Aerosols are one of the major routes of direct or surface contamination, leading to the increase of these strains during patient attendance and consequently to a higher probability of cross-infection [45,46,47]. Surfaces in clinics, as well as the attending uniform and the hands of the dentist, can be MRSA reservoirs. Patients and dental healthcare professionals can serve as hosts and reservoirs of pathogenic strains, and both can become infected. Suggested standard measures, aiming to control and reduce infection, include the disinfection of hands before and after patient attendance, as well as the use of gloves, masks, caps, glasses, and work uniforms [48,49].

The degree of MRSA/MSSA contamination detected in this study was less than 2%, which is aligned with the findings reported by Bracciale et al. (2020) [50]. The low percentage of observed contaminated GP points can probably be justified since the target was focused on the detection of a specific bacterial strain (MSSA and MRSA species). In this way, BHI and MRSA selective chromogenic culture mediums were chosen as selective ones, since the first one facilitates the growth of *S. aureus* [51], and the other is a highly reliable screening tool for the detection of MRSA [52].

To ensure direct control of potential cross-infection, the implementation of disinfection protocols is mandatory in several specific areas of dentistry. In this study, the applied chairside disinfection protocol effectively eliminated contamination in all six GP points (100%). These data are also in accordance with the findings reported by Bracciale et al. (2020) [50].

Already in 2012, the importance of GP decontamination to prevent any bacterial contamination of RCS during the filling procedure was widely recognized in endodontic practice [53]. However, due to their thermoplastic properties [54] and physical and chemical nature, GP points cannot be sterilized using physical methods such as a hot-air oven or autoclaving. To overcome these limitations, several studies have proposed a rapid chairside disinfection protocol using chemical solutions before starting the filling stage of NSRCS [53,55,56,57,58,59]. Our results support the application of a disinfection protocol, as we have demonstrated its 100% effectiveness. Nevertheless, the GP point immersion time in the disinfection solution must be kept under control, since structural changes, along with the formation of chloride crystals, have already been detected if, after disinfection with NaOCl, the GP points are not washed with sterile water or alcohol [60]. Also, prolonged immersion in NaOCl solutions for more than 1 min can lead to the loss of GP elasticity [61,62,63,64]. Besides NaOCl, many other chemical agents, such as hydrogen peroxide, chlorhexidine, ethyl alcohol, polyvinylpyrrolidone iodine, and quaternary ammonium compounds, have been tested for decontamination. Our decision to use 3% NaOCl for 60 s in this study was supported by Bracciale et al. (2020), who demonstrated the effectiveness of this protocol [50].

In future research, it would be valuable to compare the effectiveness of several chemical solutions, as well as the analysis of the total microbial contamination. This comparison should include not only the assessment of the percentage of decontamination but also the time required for GP point decontamination, along with the analysis of any potential surface alterations that may occur.

It is crucial to emphasize, among pre-graduation dentistry students, the importance of conscientiously making every effort to achieve the highest level of disinfection [65]. This includes the proper handling and storage of the GP points.

## 4. Materials and Methods

### 4.1. Sample Collection of GP Points

The typology of the analyzed GP points was limited to those most frequently used in endodontic treatments performed with manual cleaning and shaping techniques, whose taper is lower than the ones achieved with rotary systems. The main GP points had gauges ranging from K20 to K35, and the accessory ones were A and B.

A total of 315 GP points were collected from packages already in use for 1 month (used by pre-graduation students from the University Dental Clinic of the Universidade Católica Portuguesa) from six distinct International Organization for Standardization (ISO) gauges (k20, k25, k30, and k35 belonging to R&S^®^, Paris, France; and A and B belonging to Dentsply^®^, Charlotte, North Carolina, USA) (Table 2).

The inclusion criteria stipulate that all tested GP boxes were in use for at least 4 weeks, each box supported an average of 8 appointments per week, and the storage conditions adhered to the manufacturer’s specified ideal requirements for temperature and humidity.

Each GP point was inserted in one different sterile test tube (with 5 mL BHI medium) and adequately labeled. For principal GP points with the ISO gauge classification between K15 to K35, samples were coded with “1”, “2”, “3”, and “4”, corresponding to the commercial packages in test, plus the ISO codification. Similarly, samples of GP points defined as “accessory” were coded as with “A” or “B” according to their gauge.

Every day, 4 points of 4 different gauges from 2 commercial packages in test (e.g., 4 K20 points—1.1K20, 1.2K20, 1.3K20, and 1.4K20), along with 4 K25, 4 K30, 4 K35 GP points, and other exactly set from a different commercial package under examination (2.1K20, 2.2K20, 2.3K20, 2.4K20, 4 K25, 4K30 and 4K35 gauge points) were collected. This process resulted in a total of 32 GP points per day. Similarly, for accessory points, 4 points of gauge A and 4 points of gauge B were selected from two different commercial packages (“1” and “2”) labeled, for instance, “1.1A”. “1.2A”, “1.3A” and “1.4A”, “2.1A”, “2.2A”, “2.3A”, and “2.4A”, resulting in 16 accessory GP points sampled daily. This sample collection was repeated on different days until a similar number of GP points gauge was obtained. Sampling, regarding the number of GP points/gauge, was based on Bracciale et al. (2020) [50] (Table 2).

### 4.2. Evaluation of GP Points Contamination

Each collected GP point was placed in a sterile test tube containing 5 mL of BHI medium and incubated at 37 °C for 72 h. Afterwards, the tubes were examined for turbidity (microbial growth).

Sterility and growth controls were prepared and incubated at the same conditions described above (37 °C for 72 h) and examined for microbial growth by measuring the optical density at 600 nm. Uninoculated BHI medium was used as negative control (C−). As positive control (C+ SA), BHI medium inoculated with *S. aureus* ATCC 25923 was used [66].

### 4.3. Phenotypical Identification of S. aureus (MRSA/MSSA)

Positive growth tubes were selected, and each growth medium was subcultured in biplates of selective chromogenic medium—chromID^®^ MRSA/ chromID^®^ *S. aureus*—following the manufacturer’s instructions (Biomérieux, Marcy-l’Étoile, France). The green colonies were equivalent to MRSA, while the beige colonies corresponded to MSSA. Gram stain, catalase, and coagulase tests were performed according to standard procedures.

### 4.4. Molecular Identification of S. aureus (MRSA/MSSA)

The presence of MRSA strains was confirmed by the detection of the *mecA* gene by PCR [67,68,69,70,71]. Strains that tested negative for the presence of the *mecA* gene were subsequently screened for the presence of the *nuc* gene, which allows the identification of MSSA strains [71,72]. For the identification of the *mecA* gene, DNA (5 µL) was amplified in a reaction mixture containing 10 µL of 5× PCR buffer, 3 µL of MgCl_2_ 25 mM, 2 µL of dNTP mixture 10 mM, 5 µL of each primer 10 µM, and 0.25 µL of *Taq* polymerase (GoTaq^®^ Flexi DNA Polymerase, Promega, Madison, Wisconsin, USA), in a total volume of 50 µL [14]. The primers used for the identification of the *mecA* gene were 5′-GGGATCATAGCGTCATTATTC-3′ and 5′-AACGATTGTGACACGATAGCC-3′ [14]. PCR was performed as follows [14]: 10 min at 94 °C; 30 cycles of 30 s at 94 °C, 1 min at 55 °C, and 1 min at 72 °C; and a final extension of 10 min at 72 °C. For the identification of the *nuc* gene, DNA (5 µL) was amplified in a reaction mixture containing 10 µL of 5× PCR buffer, 4 µL of MgCl_2_ 25 mM, 2 µL of dNTP mixture 10 mM, 6 µL of each primer 10 µM, and 0.25 µL of *Taq* polymerase in a total volume of 50 µL [14]. The primers used for the identification of the *nuc* gene were 5′-TCAGCAAATGCATCACAAACAG-3′ and 5′-CGTAAATGCACTTGCTTCAGG-3′ [14]. PCR was performed as follows [14]: 10 min at 94 °C; 35 cycles of 30 s at 94 °C, 1 min at 58 °C, and 1 min at 72 °C; and a final extension of 10 min at 72 °C. PCR products were analyzed by 1% agarose gel electrophoresis. PCR amplification of the *mecA* gene was performed to yield a product of 527 base pairs (bp), while the amplification of the *nuc* gene resulted in a product of 255 bp. Lambda phage DNA cut with Hind III was used as molecular-weight marker in electrophoresis. Negative controls (without DNA) and positive controls (MRSA or MSSA ATCC strains) were also used in the PCR reaction.

### 4.5. Chairside Disinfection Protocol (CPD)

As described in Bracciale et al. (2020), a disinfection protocol was exclusively applied to GP points displaying contamination [50]. The implemented Cleaning and Disinfection Procedure (CPD) involved transferring the contaminated GP point from the culture tube to a 3% sodium hypochlorite (NaOCl) solution for 60 s. Subsequently, the GP point was retrieved from the disinfection immersion and rinsed with 10 mL of sterile distilled water. Afterwards, the GP point was dried with sterile gauze and placed in a new sterile tube containing BHI medium and incubation following the method previously described (Section 4.2).

### 4.6. Statistical Analysis

A statistical analysis of the results was conducted using the IBM^®^ SPSS^®^ Statistics 25.0 software (IBM Corp, released 2017, Armonk, NY, USA). Absolute and relative counts (*n* and %) were used to describe all qualitative variables. The results of dichotomic variables were conducted using the binomial test. All comparisons were performed using a 0.05 (*p* = 0.05) level of significance.

## 5. Conclusions

The risk of cross-contamination with pathogenic strains in clinics is a very serious and important problem in all health fields, and all healthcare professionals must be aware to avoid possible scenarios that can lead to infection.

Gutta-Percha points from opened pre-sterilized packages showed very low contamination by MRSA/MSSA (1.9%). The risk of transmission of pathogens, such as MRSA strains, in a dental clinic is still unknown but cannot be considered negligible.

Our study highlights the observation of bacterial growth despite minimal contamination of GP points. This underscores the importance of meticulous handling during NSRCT. Moreover, it emphasizes the critical need for consistently implementing an effective disinfection protocol. By prioritizing proper handling and adopting robust disinfection measures, we can mitigate the potential risks associated with GP-point contamination. This proactive approach is essential to ensure even higher success rates in this conservative treatment option. A recommended continuation of this study could involve the targeted analysis of other persistent endodontic pathogens, such as *Enterococcus faecalis* or *Fusobacterium nucleatum*, and *Candida albicans*. Additionally, exploring potential cross-contamination agents would be valuable. This extended analysis seeks to pinpoint and assess practical protocols that are feasible in a clinical setting in order to reduce the risk of contamination.

## Figures and Tables

**Table 1:** Results for tested contamination for the total sampling (all GP points collected) for each commercial package and gauge.

Brand and GP Point Sizes	Number of GP Points	GP Points Contaminated
Dentsply^®^ 142 (45.1%)		
A	70 (22.2%)	0
B	72 (22.9%)	3 (0.9%)
R&S^®^ 173 (54.9%)		
k20	48 (15.2%)	0
k25	46 (14.6%)	2 (0.6%)
k30	41 (13.0%)	0
k35	38 (12.1%)	1 (0.3%)
**Total**	**315 (100%)**	**6 (1.9%)**

**Table 2:** Sampling of GP points (*n* = 315) divided by brands and sizes.

Brand and GP Point Sizes	Number of GP Points
Dentsply^®^ 142 (45.1%)	
A	70 (22.2%)
B	72 (22.9%)
R&S^®^ 173 (54.9%)	
k20	48 (15.2%)
k25	46 (14.6%)
k30	41 (13.0%)
k35	38 (12.1%)

## Data Availability

The original contributions presented in the study are included in the article, further inquiries can be directed to the corresponding author.

## References

[1] Grice E.A., Kong H.H., Conlan S., Deming C.B., Davis J., Young A.C., Bouffard G.G., Blakesley R.W., Murray P.R., Green E.D. (2009). Topographical and Temporal Diversity of the Human Skin Microbiome. Science.

[2] Grice E.A., Segre J.A. (2011). The Skin Microbiome. Nat. Rev. Microbiol..

[3] Byrd A.L., Belkaid Y., Segre J.A. (2018). The Human Skin Microbiome. Nat. Rev. Microbiol..

[4] McCormack M.G., Smith A.J., Akram A.N., Jackson M., Robertson D., Edwards G. (2015). Staphylococcus aureus and the Oral Cavity: An Overlooked Source of Carriage and Infection?. Am. J. Infect. Control.

[5] Brook I. (1992). Diagnosis and Management of Parotitis. Arch. Otolaryngol. Head. Neck Surg..

[6] Spiegel R., Miron D., Sakran W., Horovitz Y. (2004). Acute Neonatal Suppurative Parotitis: Case Reports and Review. Pediatr. Infect. Dis. J..

[7] Oza N., Doshi J. (2017). Angular Cheilitis: A Clinical and Microbial Study. Indian. J. Dent. Res..

[8] Bagg J., Sweeney M.P., Wood K.H., Wiggins A. (1995). Possible Role of Staphylococcus aureus in Severe Oral Mucositis among Elderly Dehydrated Patients. Microb. Ecol. Health Dis..

[9] Subramaniam N., Muthukrishnan A. (2019). Oral Mucositis and Microbial Colonization in Oral Cancer Patients Undergoing Radiotherapy and Chemotherapy: A Prospective Analysis in a Tertiary Care Dental Hospital. J. Investig. Clin. Dent..

[10] Iatrou I.A., Legakis N., Ioannidou E., Patrikiou A. (1988). Anaerobic Bacteria in Jaw Cysts. Br. J. Oral. Maxillofac. Surg..

[11] Labriola J.D., Mascaro J., Alpert B. (1983). The Microbiologic Flora of Orofacial Abscesses. J. Oral. Maxillofac. Surg..

[12] Lewis M.A.O., MacFarlane T.W., McGowan D.A. (1990). A Microbiological and Clinical Review of the Acute Dentoalveolar Abscess. Br. J. Oral. Maxillofac. Surg..

[13] Jagadish Chandra H., Sripathi Rao B.H., Muhammed Manzoor A.P., Arun A.B. (2017). Characterization and Antibiotic Sensitivity Profile of Bacteria in Orofacial Abscesses of Odontogenic Origin. J. Maxillofac. Oral. Surg..

[14] Koukos G., Sakellari D., Arsenakis M., Tsalikis L., Slini T., Konstantinidis A. (2015). Prevalence of Staphylococcus aureus and Methicillin Resistant Staphylococcus aureus (MRSA) in the Oral Cavity. Arch. Oral. Biol..

[15] Pérez-Montarelo D., Viedma E., Murcia M., Muñoz-Gallego I., Larrosa N., Brañas P., Fernández-Hidalgo N., Gavaldà J., Almirante B., Chaves F. (2017). Pathogenic Characteristics of Staphylococcus aureus Endovascular Infection Isolates from Different Clonal Complexes. Front. Microbiol..

[16] Cheung G.Y.C., Bae J.S., Otto M. (2021). Pathogenicity and Virulence of Staphylococcus a. Virulence.

[17] Marsh P.D. (2006). Dental Plaque as a Biofilm and a Microbial Community—Implications for Health and Disease. BMC Oral Health.

[18] Coyte K.Z., Schluter J., Foster K.R. (2015). The Ecology of the Microbiome: Networks, Competition, and Stability. Science.

[19] Lamont R.J., Koo H., Hajishengallis G. (2018). The Oral Microbiota: Dynamic Communities and Host Interactions. Nat. Rev. Microbiol..

[20] Deo P.N., Deshmukh R. (2019). Oral Microbiome: Unveiling the Fundamentals. J. Oral Maxillofac. Pathol..

[21] Sudhakara P., Gupta A., Bhardwaj A., Wilson A. (2018). Oral Dysbiotic Communities and Their Implications in Systemic Diseases. Dent. J..

[22] Thomas C., Minty M., Vinel A., Canceill T., Loubières P., Burcelin R., Kaddech M., Blasco-Baque V., Laurencin-Dalicieux S. (2021). Oral Microbiota: A Major Player in the Diagnosis of Systemic Diseases. Diagnostics.

[23] Abeles S.R., Jones M.B., Santiago-Rodriguez T.M., Ly M., Klitgord N., Yooseph S., Nelson K.E., Pride D.T. (2016). Microbial Diversity in Individuals and Their Household Contacts Following Typical Antibiotic Courses. Microbiome.

[24] Swanson B.A., Carson M.D., Hathaway-Schrader J.D., Warner A.J., Kirkpatrick J.E., Corker A., Alekseyenko A.V., Westwater C., Aguirre J.I., Novince C.M. (2021). Antimicrobial-Induced Oral Dysbiosis Exacerbates Naturally Occurring Alveolar Bone Loss. FASEB J..

[25] Akhavan B.J., Khanna N.R., Vijhani P. (2023). Amoxicillin. Helicobacter Pylori.

[26] Gonçalves E., Carvalhal R., Mesquita R., Azevedo J., Coelho M.J., Magalhães R., Ferraz M.P., Manso M.C., Gavinha S., Pina C. (2020). Detection of Staphylococcus aureus (MRSA/MSSA) in Surfaces of Dental Medicine Equipment. Saudi J. Biol. Sci..

[27] Shorr A.F. (2007). Epidemiology of Staphylococcal Resistance. Clin. Infect. Dis..

[28] Pantosti A., Venditti M. (2009). What Is MRSA?. Eur. Respir. J..

[29] Bhat J.A., Tenguria R. (2014). Significance of MRSA in Nosocomial Infections. Int. J. Appl. Sci..

[30] European Centre for Disease Prevention and Control (ECDC), World Health Organization (WHO) European Region (2023). Antimicrobial Resistance Surveillance in Europe 2023–2021 Data.

[31] Contaldo M., D’Ambrosio F., Ferraro G.A., Di Stasio D., Di Palo M.P., Serpico R., Simeone M. (2023). Antibiotics in Dentistry: A Narrative Review of the Evidence beyond the Myth. Int. J. Environ. Res. Public Health.

[32] Wong J., Manoil D., Näsman P., Belibasakis G.N., Neelakantan P. (2021). Microbiological Aspects of Root Canal Infections and Disinfection Strategies: An Update Review on the Current Knowledge and Challenges. Front. Oral Health.

[33] Siqueira J.F. (2001). Aetiology of Root Canal Treatment Failure: Why Well-Treated Teeth Can Fail. Int. Endod. J..

[34] Teles A.M., Manso M.C., Loureiro S., Pina C., Cabeda J. (2013). Microorganisms: The Reason to Perform Endodontics. Microbial Pathogens and Strategies for Combating Them: Science, Technology and Education.

[35] Tabassum S., Khan F.R. (2016). Failure of Endodontic Treatment: The Usual Suspects. Eur. J. Dent..

[36] Moorer W.R., Genet J.M. (1982). Antibacterial Activity of Gutta-Percha Cones Attributed to the Zinc Oxide Component. Oral. Surg. Oral. Med. Oral. Pathol..

[37] Da Motta P.G., De Figueiredo C.B.O., Maltos S.M.M., Nicoli J.R., Ribeiro Sobrinho A.P., Maltos K.L.M., Carvalhais H.P.M. (2001). Efficacy of Chemical Sterilization and Storage Conditions of Gutta-Percha Cones. Int. Endod. J..

[38] Kayaoglu G., Gürel M., Ömürlü H., Bek Z.G., Sadik B. (2009). Examination of Gutta-Percha Cones for Microbial Contamination during Chemical Use. J. Appl. Oral. Sci..

[39] Özsezer Demiryürek E. (2012). Evaluation of Microbial Contamination of Resilon and Gutta-Percha Cones and Their Antimicrobial Activities. Afr. J. Microbiol. Res..

[40] Saeed M., Koller G., Niazi S., Patel S., Mannocci F., Bruce K., Foschi F. (2017). Bacterial Contamination of Endodontic Materials before and after Clinical Storage. J. Endod..

[41] Malmberg L., Björkner A.E., Bergenholtz G. (2016). Establishment and Maintenance of Asepsis in Endodontics—A Review of the Literature. Acta Odontol. Scand..

[42] Zahran S., Mannocci F., Koller G. (2022). Assessing the Iatrogenic Contribution to Contamination During Root Canal Treatment. J. Endod..

[43] Vishwanath V., Rao H.M. (2019). Gutta-Percha in Endodontics—A Comprehensive Review of Material Science. J. Conserv. Dent..

[44] Volgenant C.M.C., de Soet J.J. (2018). Cross-Transmission in the Dental Office: Does This Make You Ill?. Curr. Oral. Health Rep..

[45] Bernardo W.L.D.C., Boriollo M.F.G., Gonçalves R.B., Höfling J.F. (2005). Staphylococcus aureus Ampicillin-Resistant from the Odontological Clinic Environment. Rev. Inst. Med. Trop. Sao Paulo.

[46] Kobza J., Pastuszka J.S., Bragoszewska E. (2018). Do Exposures to Aerosols Pose a Risk to Dental Professionals?. Occup. Med..

[47] Hallier C., Williams D.W., Potts A.J.C., Lewis M.A.O. (2010). A Pilot Study of Bioaerosol Reduction Using an Air Cleaning System during Dental Procedures. Br. Dent. J..

[48] Williams H.N., Singh R., Romberg E. (2003). Surface Contamination in the Dental Operatory: A Comparison over Two Decades. J. Am. Dent. Assoc..

[49] Faden A. (2019). Methicillin-Resistant Staphylococcus aureus (MRSA) Screening of Hospital Dental Clinic Surfaces. Saudi J. Biol. Sci..

[50] Bracciale F., Marino N., Noronha A., Manso M.D.C., Gavinha S., Lopes Cardoso I., Pina C., Moura Teles A. (2020). Bacterial Contamination of Gutta-Percha Points from Different Brands and the Efficacy of a Chairside Disinfection Protocol. Eur. Endod. J..

[51] Wijesinghe G., Dilhari A., Gayani B., Kottegoda N., Samaranayake L., Weerasekera M. (2019). Influence of Laboratory Culture Media on in Vitro Growth, Adhesion, and Biofilm Formation of Pseudomonas aeruginosa and Staphylococcus aureus. Med. Princ. Pract..

[52] Zurita J., Mejía C., Guzmán-Blanco M. (2010). Diagnosis and Susceptibility Testing of Methicillin-Resistant Staphylococcus aureus in Latin America. Braz. J. Infect. Dis..

[53] Athiban P., Borthakur B., Ganesan S., Swathika B. (2012). Evaluation of Antimicrobial Efficacy of Aloe vera and Its Effectiveness in Decontaminating Gutta Percha Cones. J. Conserv. Dent..

[54] Siqueira J.F., Rĉças I.N., Valois C.R.A. (2001). Apical Sealing Ability of Five Endodontic Sealers. Aust. Endod. J..

[55] Stabholz A., Stabholz A., Friedman S., Heling I., Sela M.N. (1987). Efficiency of Different Chemical Agents in Decontamination of Gutta-percha Cones. Int. Endod. J..

[56] Pradeep K., Kidiyoor K., Jain P., Rao N. (2013). Chair Side Disinfection of Gutta—Percha Points—An in Vitro Comparative Study between 5 Different Agents at Different Concentrations. Endodontology.

[57] Panuganti V., Vivek V., Jayashankara C., Anilkumar S., Girish S., Nanjundasetty J. (2016). Gutta-Percha Disinfection: A Knowledge, Attitude, and Practice Study among Endodontic Postgraduate Students in India. Saudi Endod. J..

[58] Alves M.J., Grenho L., Lopes C., Borges J., Vaz F., Vaz I.P., Fernandes M.H. (2018). Antibacterial Effect and Biocompatibility of a Novel Nanostructured ZnO-Coated Gutta-Percha Cone for Improved Endodontic Treatment. Mater. Sci. Eng. C.

[59] Cardoso C.L., Kotaka C.R., Redmerski R., Guilhermetti M., Queiroz A.F. (1999). Rapid Decontamination of Gutta-Percha Cones with Sodium Hypochlorite. J. Endod..

[60] Brito S.M.S.M., de Vasconcelos R.A., Oliveira S.H.G. (2013). de Gutta-Percha Points Surface Alterations after Sodium Hypochlorite Disinfection. Braz. Dent. Sci..

[61] Valois C.R.A., Silva L.P., Azevedo R.B. (2005). Effects of 2% Chlorhexidine and 5.25% Sodium Hypochlorite on Gutta-Percha Cones Studied by Atomic Force Microscopy. Int. Endod. J..

[62] Prado M., Santos Júnior H.M., Rezende C.M., Pinto A.C., Faria R.B., Simão R.A., Gomes B.P.F.A. (2013). Interactions between Irrigants Commonly Used in Endodontic Practice: A Chemical Analysis. J. Endod..

[63] Tilakchand M., Naik B., Shetty A. (2014). A Comparative Evaluation of the Effect of 5.25% Sodium Hypochlorite and 2% Chlorhexidine on the Surface Texture of Gutta-Percha and Resilon Cones Using Atomic Force Microscope. J. Conserv. Dent..

[64] Nunes A.M., Gouvea J.P., Da Silva L. (2019). Influence of Different Disinfection Protocols on Gutta-Percha Cones Surface Roughness Assessed by Two Different Methods. J. Mater. Res. Technol..

[65] Jalan R.S., Sandeep H. Knowledge Attitude and Practice of Disinfection of Gutta Percha among Post Graduate Dental Students. Proceedings of the 2nd International Conference on Business Analytics for Technology and Security, ICBATS 2023.

[66] Bergeron M.G., Boissinot M., Huletsky A., Ménard C., Ouellette M., Picard F.J., Roy P.H. (2000). Highly Conserved Genes and Their Useto Generate Probes and Primers for Detection of Microorganisms.

[67] Datta P., Gulati N., Singla N., Vasdeva H.R., Bala K., Chander J., Gupta V. (2011). Evaluation of Various Methods for the Detection of Meticillin-Resistant Staphylococcus aureus Strains and Susceptibility Patterns. J. Med. Microbiol..

[68] Coban A.Y. (2012). Rapid Determination of Methicillin Resistance among Staphylococcus aureus Clinical Isolates by Colorimetric Methods. J. Clin. Microbiol..

[69] Stegger M., Andersen P.S., Kearns A., Pichon B., Holmes M.A., Edwards G., Laurent F., Teale C., Skov R., Larsen A.R. (2012). Rapid Detection, Differentiation and Typing of Methicillin-Resistant Staphylococcus aureus Harbouring Either MecA or the New MecA Homologue MecA(LGA251). Clin. Microbiol. Infect..

[70] Montazeri E.A., Khosravi A.D., Jolodar A., Ghaderpanah M., Azarpira S. (2015). Identification of Methicillin-Resistant Staphylococcus aureus (MRSA) Strains Isolated from Burn Patients by Multiplex PCR. Burns.

[71] Chen C., Zhao Q., Guo J., Li Y., Chen Q. (2017). Identification of Methicillin-Resistant Staphylococcus aureus (MRSA) Using Simultaneous Detection of MecA, Nuc, and FemB by Loop-Mediated Isothermal Amplification (LAMP). Curr. Microbiol..

[72] Sahebnasagh R., Saderi H., Owlia P. (2014). The Prevalence of Resistance to Methicillin in Staphylococcus aureus Strains Isolated from Patients by PCR Method for Detection of MecA and Nuc Genes. Iran. J. Public. Health.

